# Clinical utility of gene-expression profiling for tumor-site origin in patients with metastatic or poorly differentiated cancer: impact on diagnosis, treatment, and survival

**DOI:** 10.18632/oncotarget.521

**Published:** 2012-06-09

**Authors:** J. Scott Nystrom, John C. Hornberger, Gauri R. Varadhachary, Richard J. Hornberger, Hialy R. Gutierrez, W. David Henner, Shawn H. Becker, Mahul B. Amin, Michael G. Walker

**Affiliations:** ^1^ Department of Medicine, Tufts Medical Center, Boston, MA, USA; ^2^ Department of Medicine, Stanford University, Stanford, CA, USA; ^3^ Cedar Associates LLC, Menlo Park, CA, USA; ^4^ Department of Medicine, University of Texas MD Anderson Cancer Center, Houston, TX, USA; ^5^ Pathwork Diagnostics, Inc., Redwood City, CA, USA; ^6^ Department of Pathology and Laboratory Medicine, Cedars-Sinai Medical Center, Los Angeles, CA, USA

**Keywords:** cancer of unknown primary, diagnosis, genomics, clinical utility, tissue of origin

## Abstract

**PURPOSE:**

The primary tissue-site origin in over 4% of cancers remains uncertain despite thorough clinicopathological evaluation. This study assessed the effect of a Food and Drug Administration-cleared 2,000-gene–expression-profiling (GEP) test on primary tissue-site working diagnoses and management for metastatic and poorly differentiated cancers.

**METHODS:**

Clinical information was collected from physicians ordering the GEP test for patients with difficult to diagnose cancers. Endpoints included diagnostic procedures, physicians’ working diagnoses and treatment recommendations before and after GEP result availability, and physician reports of the test's usefulness for clinical decision making. Patient date of death was obtained, with a minimum of one year follow-up from date of biopsy.

**RESULTS:**

Sixty-five physicians participated in the study (n=107 patients). Before GEP, patients underwent 3.2 investigations on average (e.g., radiology, endoscopy). Ten immunohistochemistry tests were used per biopsy (SD 5.2). After GEP testing, physicians changed the primary working diagnosis for 50% of patients (95% CI: 43%,58%) and management for 65% of patients (95% CI: 58%,73%). With GEP results, the recommendation for guideline-consistent chemotherapy increased from 42% to 65% of patients, and the recommendation for non-guideline-consistent regimens declined from 28% to 13%. At last follow-up, 69 patients had died, and median survival was 14.0 months (95% CI: 10.2,18.6). Thirty-three percent of patients were alive at 2 years.

**CONCLUSION:**

In patients with difficult-to-diagnose cancers, GEP changed the working diagnosis and management for the majority of patients. Patients for whom the GEP test was ordered had longer median survival than that historically reported for patients enrolled in treatment trials for cancer of unknown primary.

## INTRODUCTION

More than 4% of patients diagnosed with cancer present with metastases whose primary origin remains uncertain despite thorough clinical and pathological evaluation [1]. Other patients who were previously diagnosed and treated for cancer may develop a tumor in a new site whose new primary site is unknown. Complete characterization of the primary tissue site of origin in such cases remains clinically challenging.

Guidelines recommend that patients with occult metastatic cancers undergo a thorough evaluation, including a complete history, physical examination, complete blood count, urinalysis, serum chemistries, histologic evaluation, chest radiograph, computed tomography, magnetic resonance imaging, and immunohistochemistry (IHC) studies [2-6]. The 2007 Ad-Hoc Committee on Immunohistochemistry Standardization addressed several potential “deficiencies” in the consistency, reproducibility, quality assurance, concordance, validation, and results reporting of IHC studies [7]. Even with adoption of such recommendations, full characterization of the tumor-site origin may remain elusive [8].

Gene-expression profiling (GEP) for tissue of origin of biopsy material represents a newer method for characterizing the tumor site of origin. In June 2010, the US Food and Drug Administration cleared the Pathwork® Tissue of Origin Test Kit for formalin-fixed, paraffin-embedded tissue samples (Pathwork Diagnostics, Inc.; Redwood City, CA, USA). The processing laboratory has Clinical Laboratory Improvement Amendments certification. This microarray, reagent, and analytics kit compares the similarity of tumor specimens to 15 cancer types representing 58 morphologies. GEP results provide similarity scores which range from 0-100 and indicate the most likely primary site from among a panel of 15 tissue types. The sensitivity and specificity of the GEP test cannot be measured directly against CUP specimens because there is no gold standard for their primary site. In a large multi-site blinded validation study, GEP has been shown to identify the primary site of metastatic tumors with sensitivity equal to 89% (Positive Percent Agreement) and specificity equal to 99% (Negative Percent Agreement) [9]. GEP provides clinically useful information for metastatic cancers with poorly differentiated tumors that complements information from traditional sources, such as clinical history and physical examination, imaging studies, histology, and IHC results [10-12].

The principles for appraising new molecular diagnostics have been evolving since the sequencing of the human genome [13, 14]. For a laboratory test to have the potential to improve clinical outcomes, awareness of the test results should lead to appropriate changes in management decisions by patients and providers [13, 14].

This study assessed the impact of the GEP test results, when used as an adjunct to clinicopathological evaluation, on tissue-site working diagnosis, subsequent management decisions, and survival.

## RESULTS

We invited 316 physicians to participate in this study. Of these, 152 did not respond to recruitment efforts, 82 declined participation, and 9 consented to participate but did not finalize data entry. Eight physician reports failed to meet inclusion criteria. The final sample consisted of 65 physicians who had ordered and received a GEP test for 107 patients between July 2009 and December 2009.

Most physicians in the study (62%) were part of a group practice and 57 (88%) were board-certified in medical oncology (Table 1). Sixty percent reported seeing at least six patients per year with difficult-to-diagnose tumors. Sixty-one (57%) patients were women; mean patient age was 64 (standard deviation [SD] 12), and 54 (50%) patients were at least 65 years old. Of the 91 patients with a reported Eastern Cooperative Oncology Group performance status score, 87 (81%) had a score of 2 or less.

**Table 1:** Physician and patient characteristics

	N, mean (SD)	%
**Physicians**		
Total respondents	65	
Female	12	18%
Practice setting*		
Group-based	45	62%
Hospital-based	12	16%
Academic	9	12%
Solo	5	7%
Department of Veterans Affairs	1	1%
Other	1	1%
Board certification		
Medical oncology - hematology oncology	41	63%
Medical oncology	16	25%
Other^†^	8	12%
Years since completing training, mean (SD)	13 (11)	
Number of patients seen per year with difficult-to-diagnose cancer		
0-5	26	40%
6-10	25	38%
11-20	10	15%
>20	4	6%
**Patients**	**107**	
Female	61	57%
Age		
All, mean (SD)	64 (12)	
Age 65 and older	54	50%
Race/ethnicity		
White	95	89%
African American	4	4%
Hispanic, Latino or Spanish heritage	4	4%
Asian American	2	2%
Other	2	2%
Eastern Cooperative Oncology Group performance status		
0	31	29%
1	35	33%
2	21	20%
3	4	4%
Not reported	16	15%

* Total is greater than 65 because 5 practices were both academic and hospital-based, 2 practices were both hospital-based and group-based, 1 practice was solo and academic, and 1 practice was group-based and “Other”, specified as a “cancer center”.

† Other: gynecologic oncology, obstetrics and gynecology, orthopedic surgery and oncology, thoracic surgery, general surgery, and infectious disease.

Abbreviations: SD - standard deviation.

The tissue samples submitted for GEP testing came from 19 biopsy sites. The most common sites were lymph node (21%), soft tissue (20%), liver (18%), lung (9%), bone (7%), and brain (5%). Participating physicians ordered 343 imaging or endoscopic investigations, for an average of 3.2 investigations per patient (Table 2). All patients had an imaging study; all but 1 patient had imaging with a magnetic resonance imaging, computed tomography, or positron emission tomography. That patient had ovarian cancer, which was imaged with ultrasound. The most common test, ordered for 85% of patients, was a computed tomography scan. Overall, imaging and endoscopic tests detected a tumor in more than 19 sites, with the most common sites being lymph node, liver, and lung. Physicians ordered 147 IHC tests, with a mean of 9.9 (SD 5.2) IHCs per biopsy.

**Table 2:** Pathology and imaging or endoscopic investigations

	N	%
**Imaging tests and other investigations**		
CT scan	91	85%
PET	74	69%
Upper endoscopy	36	34%
MRI	33	31%
Colonoscopy	33	31%
Bone scan	31	29%
Mammography	30	28%
Ultrasound (any)	15	14%
**Pathology – number of IHC tests per biopsy**		
0-5	22	21%
6-10	39	36%
11-15	27	25%
16-20	16	15%
>20	3	3%

Abbreviations: CT- computerized tomography; IHC -immunohistochemistry; MRI - magnetic resonance imaging; PET - positron emission tomography.

**Figure 1:**
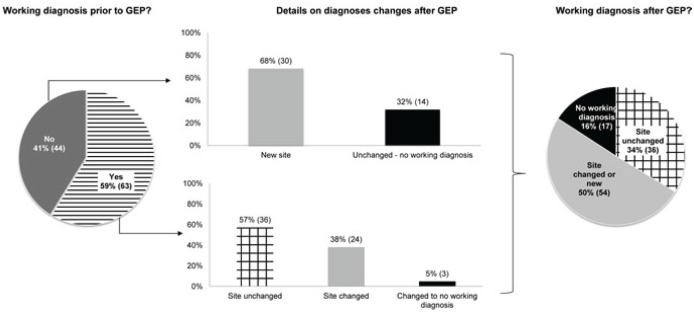
Proportion of site-specific diagnoses before and after GEP *Abbreviations: GEP - gene-expression profile*.

Before receiving the GEP test results, physicians reported having no working diagnosis for 41% (44) of patients (Figure 1). The most common pretest tumor-site working diagnoses reported for the other 59% (63) of patients were lung (9), cholangiocarcinoma (7), breast (6), colon (5), ovarian (5), gastric (4), and pancreas (3).

After receiving the GEP test results, physicians changed the primary working diagnosis site for 54 patients (50%, 95% CI: 43%, 58%; p<0.0001). The number of patients with no working diagnosis declined from 44 to 17 (25% difference, 95% CI: 15%, 36%; p<0.0001; Table 3). The tissue-site working diagnosis remained unchanged in 37 (35%) cases. For example, of the 10 cases that specified lung as the initial tissue-site working diagnosis, 7 remained specified as lung after GEP test; the tissue-site working diagnosis similarly remained unchanged before and after GEP testing in 3 of 7 cholangiocarinomas, 5 of 6 breast cancers, 2 of 5 colon cancers, 5 of 5 ovarian cancers, and 1 of 3 pancreatic cancers. More cancers had a reported site working diagnosis of colon, lung, pancreas, or breast after GEP. In contrast, the number of patients with a cholangiocarcinoma working diagnosis decreased from seven to three after the test, and the number of gastric cancer working diagnoses dropped from four to two. The most common GEP results were colorectal (19%), breast (14%), sarcoma (14%), lung (11%), ovarian (11%), and pancreas (10%) tissue types. The most common working diagnoses after GEP were lung (13%), colorectal (11%), pancreas (9%), and ovarian (8%) cancer.

**Table 3:** Tissue type working diagnoses before and after GEP results

Tissue type	Physician's pre-GEP diagnosis		Physician's post-GEP diagnosis		Percent change
	*N*	%	*N*	%	
No working diagnosis	44	41%	17	16%	−25%
Anal	1	1%	1	1%	0%
Appendix	1	1%	0	0%	−1%
Bladder	1	1%	2	2%	1%
Breast	6	6%	8	7%	2%
Cervical	1	1%	1	1%	0%
Cholangiocarcinoma	7	7%	3	3%	−4%
Colorectal	5	5%	12	11%	7%
Esophagus	1	1%	0	0%	−1%
Extragonadal germ cell	1	1%	1	1%	0%
Extraosseous Ewing's sarcoma	1	1%	0	0%	−1%
Gastric	4	4%	2	2%	−2%
Gastrointestinal	1	1%	0	0%	−1%
Head and neck	4	4%	3	3%	−1%
Kidney	1	1%	3	3%	2%
Liver	0	0%	4	4%	4%
Lung	10	9%	14	13%	4%
Melanoma	2	2%	2	2%	0%
Ovarian	5	5%	9	8%	4%
Pancreas	3	3%	10	9%	7%
Pancreatic neuroendocrine	1	1%	1	1%	0%
Pancreatobiliary	1	1%	2	2%	1%
Parotid gland	0	0%	1	1%	1%
Prostate	1	1%	0	0%	−1%
Renal	1	1%	0	0%	−1%
Sarcoma	0	0%	6	6%	6%
Testicular germ cell	0	0%	0	0%	0%
Upper GI adenocarcinoma	1	1%	0	0%	−1%
Urethral	0	0%	1	1%	1%
Urothelial	2	2%	3	3%	1%
Uterine	1	1%	1	1%	0%

Abbreviations: GEP - gene-expression profiling; GI - gastrointestinal.

Overall, for 70 patients (65%, 95% CI: 58%, 73%; p<0.0001) physicians changed one or more management recommendation after GEP (Table 4). For 17 patients, physicians did not change the primary working diagnosis but changed management recommendations. Prior to the test, physicians reported recommending 44 different chemotherapy drug combinations for 75 patients and no chemotherapy for 32 patients. Carboplatin plus paclitaxel was recommended in 26 patients. Physicians changed their recommended chemotherapy regimens after receiving GEP results for 58 patients (54%, 95% CI: 46%, 62%; p<0.0001, Table 4). With GEP results, the recommendation for chemotherapy regimens that are consistent with guidelines for the final tissue-type working diagnosis increased by 23%, from 42% to 65% of patients, and the recommendation for regimens not consistent with guidelines for the final tissue-type working diagnosis declined by 15%, from 28% to 13%.

**Table 4:** Changes in management*

	N	%
Any aspect of management	70	65%
Chemotherapy, change	58	54%
Radiation therapy, change^†^	27	25%
Further investigations^‡^, increase	18	17%
Hospice, increase	14	13%
New surgeries, increase	5	5%

* Patients may have had more than one aspect of management change after gene expression profiling test.

† A net 19 fewer patients received radiation therapy.

‡ 6 referrals, 4 immunohistochemistry tests, 2 repeated biopsies, 2 computed tomography scans, 1 immunohistochemistry test and referral, 1 repeated positron emission tomography scan, 1 colonoscopy and excision, and 1 consult and cytoscopy. Of these 18 patients who underwent further investigations, 7 patients’ diagnoses changed after the physician received GEP results, and 10/15 physicians stated that their diagnosis was most influenced by GEP (3 did not provide an answer).

**Figure 2:**
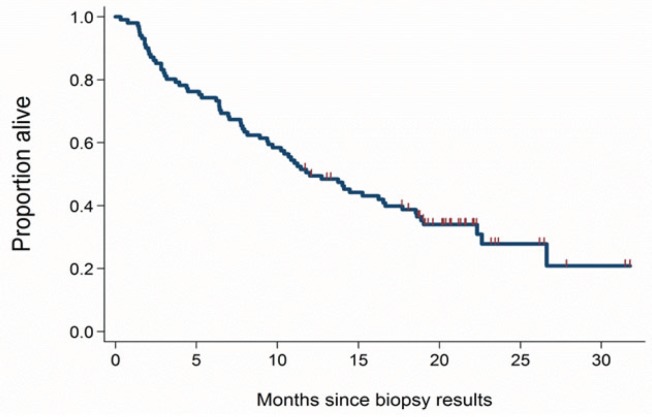
Survival from time of biopsy Median survival was 14.0 months from time of biopsy.

Median survival from time of the biopsy was 14.0 months (95% CI: 10.2, 18.6), with 33% of patients alive at 2 years (95% CI: 24%, 44%) and 30% of patients alive at 3 years (95% CI: 20%, 41%; Figure 2). Median time from biopsy to requisition of GEP was 1.0 months (95% CI: 0.9, 1.3). The most common final working diagnoses for patients alive at 3 years were breast (7), lung (7), colorectal (5), ovarian (4), and no working diagnosis (4). Prior to GEP test results, for the 61% of the patients for whom physicians had identified a pretest primary site, physicians ranked conventional pathology (including IHC testing) as the most influential factor for diagnosis in 53% of patients. After receiving the GEP results, for these patients, physicians cited the GEP test as the most influential factor for diagnosis in 60% of the patients; physicians cited conventional pathology as the most influential factor for diagnosis in 23% of all patients.

When asked whether the test changed their approach to treatment, physicians reported agreeing or strongly agreeing for 52% of patients. Two-thirds of physicians agreed or strongly agreed that the test results were clinically useful in the care of their patient. Compared with leaving the tissue-site working diagnosis unspecified after GEP testing, being able to specify a tissue-site working diagnosis or not changing a previously-specified tissue site was associated with agreement or strong agreement that the test was clinically useful. Changes in surgical plans, orders for more investigations, or both were associated with agreement that the GEP test was clinically useful.

Physicians stated that the GEP test was helpful because it (1) provided guidance in choosing more targeted therapy, (2) confirmed a working diagnosis, (3), helped alleviate patient anxiety, (4) led to a more appropriate further workup or triaging to a more appropriate specialist, or (5) helped rule out the least likely possible diagnoses. Of physicians who did not agree that the GEP test was helpful, 10 (9%) reported that the likely diagnosis was not on the report panel and 6 (6%) did not believe the results.

## DISCUSSION

This study measured the influence of GEP testing on tissue-site working diagnosis and management decisions for cancers with a difficult-to-diagnose primary. Participating physicians’ use of imaging tests, other clinical investigations, and IHCs to determine the tumor site of origin appeared consistent with guidelines. Despite these physicians’ thorough clinicopathological evaluations, the GEP test altered the tissue-site working diagnosis in 50% of patients and significantly reduced the proportion of unspecified working diagnoses. These results confirm those of a previous analysis that used modeling to measure the impact of the GEP on working diagnosis and treatment for a series of 300 cases [15].

This study did not measure the effects of GEP on cancer control/progression, patient survival, or quality of life directly. However, several physicians reported changing treatment protocols because they believed having a more accurate tumor site of origin would direct organ-specific therapeutic interventions and, in turn, potentially improve patient survival and quality of life. Survival for patients with cancer of unknown primary is generally poor; median survival without chemotherapy is 3 to 5 months and less than 20% of patients are still alive at 2 years [2, 6, 16-18]. Systematic reviews of studies involving cancer of unknown primary suggest that chemotherapy may increase survival to approximately 9 months. Newer chemotherapeutic regimens for specific tissue diagnoses have demonstrated increased survival in patients with advanced stage cancer (e.g., FOLFOX for colon and sunitinib for kidney cancer). Whether the survival results observed in this study are a result of a change in management due to more specific tissue diagnosis remains a question for future research.

Results of this study should be interpreted in light of possible limitations. First, physicians who ordered the test and therefore were eligible to participate in the study may differ from physicians who have not ordered the test. For example, the physicians in this study might be more comfortable than other physicians with using new technologies, have more financial incentives for using such technologies, have access to more pathology resources, or be more willing to acknowledge limitations of existing diagnostic methodologies. Second, many physicians did not respond or refused to participate in the study; these physicians may hold different views from those who did participate about the test's usefulness. These factors may affect the generalizability of the findings. However, biopsy sites of specimens in this study were representative of all samples processed by the laboratory, suggesting that study patients were representative of patients for whom GEP is ordered. Third, physicians were asked to refer to data in medical records; our findings are only as robust as the quality of the participants’ documentation and recall. Fourth, the scope of the study was limited to determining the extent to which, if at all, the GEP test influenced the tumor site-of-origin working diagnosis and subsequent management plan.

This study confirms findings in the literature that patients with metastatic cancers with uncertain primary undergo multiple imaging and laboratory tests, a process referred to as a “diagnostic odyssey” [19, 20]. This odyssey delays treatment for patients and adds costs to the healthcare system. With appropriate usage, diagnostic tools such as the GEP have a potential to help reduce this burden [21, 22].

Evidence of an impact on diagnostic and management decisions is now considered an essential requirement for assessing the clinical utility of new diagnostics [13, 14]. This study suggested that for a majority of patients with a primary tumor that was difficult to diagnose, uncertain, or both, GEP testing influenced both tissue-of-origin site working diagnosis and treatment recommendations. Moreover, the observed median survival in this cohort was longer than that reported for patients with cancer of unknown primary enrolled in treatment trials [23-25]. As suggested by this study, the GEP test has clinical utility because it can increase physician confidence in cancer diagnoses, assist physicians in targeting therapies, and increase compliance with treatment guidelines.

## MATERIALS AND METHODS

An institutional review board approved all aspects of this retrospective, observational study (Quorum Review Inc., Seattle, WA).

Physicians were eligible for inclusion in the study if they ordered the GEP test between July and December 2009, the test was completed, and they received a report of the results (similarity scores to 15 tissues of origin). Physicians were excluded if they were not the treating physician (e.g., if they were a pathologist). Test reports were ineligible if the patient's sample could not be analyzed, the patient was younger than 18 years, the patient died before the physician received the test results, or the test was completed prior to the study start date. The sponsor (Pathwork® Diagnostics, Redwood City, CA USA) provided a dataset of eligible physicians to the research center, which enrolled and conducted follow-up interviews with all participating physicians. This center received no patient identifying information.

The list of eligible physicians was randomly ranked and physicians were selected into a pilot group and four subsequent recruitment groups based on each physician's rank. Members of each group received a recruitment package containing a cover letter explaining the study, the consent form, a list of eligible GEP test report numbers, and online links to the consent form and data-collection instruments. Physicians who chose to participate signed an informed consent form and completed a case report form on patient demographics and biopsy site, diagnostic studies and results before receiving the GEP test results, working and differential diagnosis before and after GEP, functional status before and after GEP, intended management before GEP, management after GEP, patient's current status, perceived clinical usefulness of GEP, and physician demographics. Pathwork® Diagnostics provided de-identified GEP test results; the tissue type with the highest GEP similarity score was used for analysis.

We also obtained de-identified Social Security data on whether each patient had died and the date of death. We used this data to calculate duration of survival from the time that the test results were reported.

## DATA ANALYSIS

The study's primary endpoints were (1) the proportion of patients whose tissue-site working diagnosis changed after GEP testing and (2) the proportion whose cancer-specific treatment changed after GEP testing. Secondary endpoints included physician perceptions of the test's clinical utility and of patient responses to cancer-specific therapy.

In our multi-level analysis, the unit of analysis was the patient case for primary endpoints and the physician for secondary endpoints. We based the sample size for patient cases on exact one-sided Clopper-Pearson confidence intervals (CIs) [26, 27]. A sample size between 76 and 137 patients was deemed appropriate to produce a one-sided 95% lower-limit CI with a difference in pre-/post-working diagnosis within 6% to 8%.

All study variables are summarized using descriptive statistics. The proportional change in tissue-site working diagnoses and management was assessed with a one-sample McNemar's test [28].

Ordered logistic regression was used to estimate the effect of changes in tissue-site working diagnosis and in management on physicians’ reports of the GEP test's clinical usefulness. After we controlled for age, gender, number of IHC tests, and number of imaging tests, we evaluated the proportional change in working diagnoses and management with a two-sided test. Survival from time of biopsy was estimated using the Kaplan-Meier product-limit estimator; censoring indicates alive on June 14, 2011. Analyses were conducted in Microsoft® Excel (Microsoft® Corporation, Seattle, WA USA) and STATA v9.2 (STATA Corp®, College Station, TX USA) [28]. Two researchers independently determined whether to classify each chemotherapy regimen as guideline-consistent or not guideline-consistent based on the regimens recommended in National Comprehensive Cancer Network and Up-To-Date guidelines for metastatic and/or poorly differentiated cancers, and according to the physician's final tissue-site working diagnosis [29, 30].
